# Upper limb replantation: functional disability and quality of life challenges

**DOI:** 10.3389/fresc.2026.1648169

**Published:** 2026-04-30

**Authors:** Andrea Bueno-de la Fuente, Sandra Núñez-Rodríguez, Raquel de la Fuente-Anuncibay, Miguel Eugenio Estefanía-Díez, Endika Nevado-Sanchez, Jerónimo Javier González-Bernal

**Affiliations:** 1 Santa Clara Health Center, Burgos, Spain; 2 Department of Health Sciences, Universidad Isabel I, Burgos, Spain; 3 Department of Education, University of Burgos, Burgos, Spain; 4 Reconstructive and Aesthetic Plastic Surgery Service, Hospital Universitario de Burgos, Burgos, Spain; 5 Department of Health Sciences, University of Burgos, Burgos, Spain

**Keywords:** disability perception, functional recovery, quality of life, rehabilitation outcomes, return to work, upper limb replantation

## Abstract

**Background:**

Upper limb replantation, while surgically complex, demands a multidimensional assessment of functional recovery, subjective satisfaction, and quality of life beyond mere anatomical survival of the limb.

**Objective:**

This study aimed to evaluate the functional outcomes, health-related quality of life, and perceived satisfaction of patients after upper limb replantation, with special attention to the impact of surgical reintervention, rehabilitation, and return to work.

**Methods:**

We conducted a cross-sectional observational study including 62 patients treated at a referral center between 2021 and 2023. Patients were assessed using validated instruments (DASH, SF-12, Russell test) alongside clinical and sociodemographic data. Statistical analyses explored associations between reintervention, functionality, quality of life, and work reintegration.

**Results:**

Nearly half of the patients required reintervention, which was significantly associated with greater functional disability in occupational and recreational contexts and lower subjective use of the hand in daily activities. Return to work was linked to better functional scores, although quality of life measures showed limited association with functional outcomes.

**Conclusion:**

Surgical reintervention and failure to return to work are key factors associated with perceived disability after upper limb replantation. These findings highlight the need for individualized, multidisciplinary follow-up protocols that integrate functional, psychological, and occupational rehabilitation strategies to optimize patient recovery.

## 1 Introduction

The success of upper limb replantation is not measured solely by the viability of the replanted segment. In clinical practice, achieving tissue perfusion and anatomical union is merely the starting point (1). The real challenge arises afterward: restoring functionality, reintegrating the patient into work and social environments, and achieving an acceptable quality of life. These deeply subjective factors do not always correlate with the objective surgical outcome or the expectations of the medical team (2, 3).

Upper limb replantation involves the surgical reattachment of an amputated segment, including microsurgical reconnection of arteries, veins, nerves, tendons, and bone structures (4). It is a technically demanding procedure that requires rapid multidisciplinary coordination, specialized hospital infrastructure, and prolonged rehabilitative follow-up (5–7). Nevertheless, even when limb survival is achieved, functional sequelae may be significant. Surgical reintervention, which occurs in a substantial proportion of patients, represents a critical factor that may negatively affect both functional and emotional recovery (1, 8, 9). Postoperative complications such as infections, partial necrosis, stiffness, or tendon retraction may compromise technical outcomes and increase perceived distress and the risk of persistent disability (1, 10–12).

After replantation, many patients face lasting functional limitations that affect not only daily activities but also their ability to return to work (9, 13, 14). This issue becomes especially relevant in cases requiring surgical reintervention, as these procedures can alter anatomical recovery and are associated with greater perceived disability, particularly in work-related or recreational tasks. Furthermore, factors such as the laterality of the affected limb, the type of rehabilitation received, and self-reported quality of life may act as modulators of recovery and should be considered comprehensively(1, 3).

Despite these aspects, the literature remains largely focused on objective surgical variables and microvascular success rates, with limited multidimensional approaches that integrate the patient's functional, psychosocial, and subjective perspectives. Tools such as the DASH questionnaire, the SF-12, and the Russell test provide complementary assessments of perceived disability, health-related quality of life (HRQoL), and personal satisfaction with functional recovery (15–17) (19–21). However, few studies have combined these scales in the context of replantation or examined the influence of factors such as reintervention, rehabilitation, or actual limb use.

In this context, the present study aims to evaluate functionality, quality of life, and subjective satisfaction in patients undergoing upper limb replantation, with special emphasis on the impact of surgical reintervention, the rehabilitation received, and the return to work activity. By combining validated instruments with clinical and sociodemographic variables, this study seeks to provide evidence that supports a more comprehensive understanding of recovery and facilitates clinical decision-making and individualized postoperative follow-up planning.

## 2 Materials and methods

### 2.1 Study design and participants

An observational, cross-sectional study with a descriptive and inferential approach was conducted to analyze clinical, functional, sociodemographic, and subjective variables in patients who underwent upper limb replantation.

**Figure 1:**
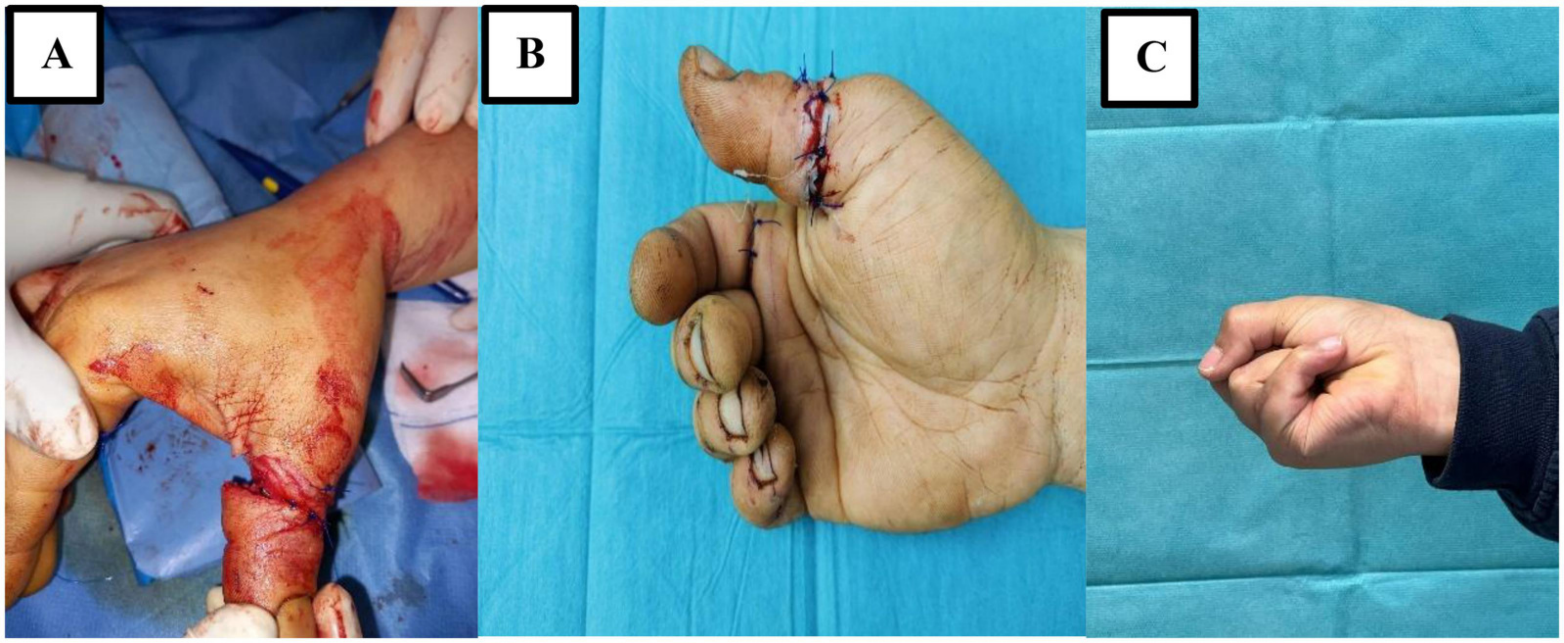
Representative images of upper limb replantation. Representative example of thumb replantation. The cohort included replantations at multiple anatomical levels, including other single digits, multiple digits, hand, and arm replantations. **(A)** Intraoperative view showing traumatic amputation with soft tissue and vascular injury. **(B)** Immediate postoperative result after replantation, demonstrating microvascular repair and soft tissue closure. **(C)** Postoperative functional outcome showing active hand flexion during follow-up.

The sample consisted of 62 patients who underwent surgical procedures at the University Hospital of Burgos between 2021 and 2023. The surgical procedure involved microvascular anastomosis, tendon repair and soft tissue closure (Figure 1), as illustrated by intraoperative images. Replantations involved different anatomical segments depending on the injury pattern and surgical indication, including thumb, other single digits, multiple digits, hand, and arm replantations. A non-probability sampling method was used, targeting patients who met the inclusion criteria and voluntarily agreed to participate in the study.

Inclusion criteria were: (1) having undergone upper limb replantation during the specified period, (2) retaining sufficient cognitive abilities to complete the questionnaire, and (3) providing informed voluntary consent. Upper limb replantations at different anatomical levels were included. The anatomical level of replantation was categorized according to the most proximal segment involved and classified as thumb, other single digit, multiple digits, hand, or arm replantation. No exclusion criteria based on amputation level were applied in order to reflect real clinical practice. Exclusion criteria included individuals without access to technological means of communication (telephone, email, or mailing address), and those unable to effectively participate in the evaluation.

### 2.2 Procedure

Patient medical records were reviewed to verify inclusion criteria. Afterwards, patients were contacted by phone to request informed consent, explain the study objectives, and offer two modes of participation: an online questionnaire (Microsoft Forms) or a structured personal interview. The estimated response time ranged from 30 to 40 min. Participants were assured of their right to withdraw at any time and of the anonymity and confidentiality of all collected data. Only fully completed questionnaires were included in the analysis.

The study was approved by the Ethics Committee of the University Health Complex of Burgos (Ref. CEIm 2949) and was conducted in accordance with the principles of the Declaration of Helsinki and Spanish Organic Law 3/2018 on the Protection of Personal Data and Guarantee of Digital Rights. All participants provided informed consent prior to participation. All intraoperative and clinical images were obtained with patient consent, and all identifying information was removed to preserve patient anonymity.

### 2.3 Assessment instruments and variables

An *ad hoc* questionnaire was used to collect sociodemographic variables (sex, age, family status, current and previous occupation), clinical variables (date of surgery, mode of transport to hospital, affected limb, dominant laterality, injury mechanism, smoking habits, comorbidities such as diabetes or anticoagulant treatment, allergy history, participation in rehabilitation, need for reintervention, and wound healing progress), and three validated scales to evaluate functionality, quality of life, and subjective outcome perception:

- **DASH (Disabilities of the Arm, Shoulder, and Hand):** a 30-item self-administered questionnaire that assesses overall upper limb functionality. It provides a total score transformed into a scale from 0 (no disability) to 100 (maximum disability). It is a validated tool with high reliability and clinical sensitivity (15, 18).
- **SF-12 version 2:** a generic health-related quality of life instrument consisting of 12 items across eight dimensions, grouped into two components: Physical Component Summary (PCS) and Mental Component Summary (MCS). It assesses the patient's perceived health status over the past four weeks using Likert-type response options (17, 19).
- **Russell Test:** a subjective scale for evaluating surgical outcomes. It includes six items exploring perceived functionality, return to work, comparison with prosthesis, recommendation of the procedure, use of the limb in daily activities, and overall satisfaction. Higher scores indicate lower perceived distress and better acceptance of the outcome (9). The Russell questionnaire was used as an item-level descriptive measure of subjective outcome (e.g., perceived use in daily activities, satisfaction, recommendation). It has been specifically applied in upper limb replantation studies and allows direct assessment of patient-perceived functional use following replantation. To our knowledge, the Russell test has not been formally validated as a psychometric patient-reported outcome measure (PROM) in this target population; therefore, results derived from this instrument were presented descriptively and interpreted with caution.

### 2.4 Statistical Analysis

Statistical analysis was conducted using IBM SPSS Statistics, version 26.0. Non-parametric tests were used due to the nature of the data and sample size. To explore associations between continuous and ordinal variables, Spearman's correlation coefficient was applied. Group comparisons were performed using the Mann–Whitney U test, suitable for independent samples without normality assumptions. Associations between categorical variables were analyzed using the Chi-square test, incorporating adjusted residuals to identify cells with significant contributions to the association. A statistical significance level of *p* < 0.05 was established for all analyses. Missing or incomplete responses were handled using pairwise deletion, allowing analyses to be performed using all available data for each variable or comparison. Because some instruments included optional modules (e.g., DASH work and sport/music) and some clinical variables were missing, the effective sample size varied across analyses. The corresponding sample size for each comparison is indicated in the tables.

## 3 Results

### 3.1 Clinical and sociodemographic characteristics of the sample

The sample consisted of 62 patients ranging in age from 6 to 83 years, with a mean age of 51.8 years (SD = 17.3). Table 1 presents the frequencies and percentages of the study's sociodemographic variables. Regarding sex, males predominated, accounting for 87.7% (*n* = 55), while females represented 11.3% (*n* = 7). A total of 32.3% of patients had injuries affecting their dominant limb.

**Table 1:** Sociodemographic and clinical characteristics of the sample^a^.

Sociodemographic and Clinical Characteristics		N (%)
Gender	Male	55 (87.7%)
	Female	7 (11.3%)
Affected dominant limb	Yes	20 (32.3%)
	No	31 (50%)
Level of replantation	Thumb	7 (11.3%)
	Other single digit	3 (4.8%)
	Multiple digits	1 (1.6%)
	Hand	43 (69.4%)
	Arm	8 (12.9%)
Smoker	Yes	13 (21%)
	No	49 (79%)
Diabetes Mellitus	Yes	5 (8.1%)
	No	56 (90.3%)
Antiplatelet agents (e.g., aspirin) and anticoagulants (e.g., warfarin)	Yes	10 (16.1%)
	No	52 (83.9%)
Allergies	Yes	8 (12.9%)
	No	54 (87.1%)
Rehabilitation	Yes	55 (88.7%)
	No	7 (11.3%)
Type of center where rehabilitation was received	Public	24 (38.7%)
	Private	23 (37.1%)
	Both	9 (14.5%)
Cause of injury	Domestic accident	11 (17.7%)
	Work accident	36 (58.1%)
	Traffic accident	2 (3.2%)
	Other	13 (21.0%)
Required reintervention	Yes	29 (46.8%)
	No	33 (53.2%)
Wound healing complications	Yes	6 (9.7%)
	No	56 (90.3%)
Resumption of work activity	Yes	32 (51.6%)
	No	30 (48.8%)

^a^Sample size varies across variables due to missing data, including affected dominant limb (*n* = 51), diabetes (*n* = 61), wound-healing complications (*n* = 61), and rehabilitation setting (*n* = 56).

Regarding the level of replantation, injuries included thumb replantations (11.3%), other single-digit replantations (4.8%), multiple-digit injuries (1.6%), hand-level replantations (69.4%), and arm-level replantations (12.9%).

In terms of health-related habits, 21% were active smokers and 8.1% had a history of diabetes mellitus. Additionally, 16.1% were undergoing antiplatelet or anticoagulant treatment, and 12.9% reported a known allergy.

Regarding the recovery process, 87.1% of patients received rehabilitative treatment after surgery, with 38.7% treated in public centers, 37.1% in private facilities, and 14.5% in both types of healthcare institutions.

As for the injury mechanism, the majority of cases were due to work-related accidents (58.1%), followed by domestic accidents (17.7%), other miscellaneous causes (21.0%), and traffic accidents (3.2%).

From a postoperative clinical perspective, 46.8% of patients required at least one surgical reintervention, and 9.7% experienced wound healing complications. Finally, 51.6% of participants reported returning to work (*n* = 32), whereas 48.4% had not resumed work at the time of assessment (*n* = 30). This variable reflects self-reported work resumption status obtained from the Russell test. Some participants were not engaged in occupational activity prior to injury due to age or other circumstances.

### 3.2 Inferential analysis

#### 3.2.1 Relationship between functionality and quality of life

Spearman correlation analysis was conducted to explore associations between DASH questionnaire scores (total and optional modules) and the physical and mental components of the SF-12.

Results revealed a moderate positive correlation between the total DASH score and both the work and sport/music modules, indicating greater perceived disability in occupational and recreational activities.

No statistically significant associations were observed between the total DASH score and either the physical or mental components of the SF-12.

The DASH work module showed a significant association with the physical component of health-related quality of life, whereas no association was observed with the mental component. Similarly, the sport/music module did not demonstrate significant associations with either physical or mental health-related quality of life.

The published minimally clinically important difference (MCID) for the DASH questionnaire has been reported as 10.83 points, which provides a reference for interpreting whether observed differences may be clinically meaningful beyond statistical significance. Detailed correlation coefficients are presented in Table 2.

**Table 2:** Correlations between DASH questionnaire scores and SF-12 physical and mental components.

Variables	DASH Total	DASH Work	DASH Sports and Music	SF Physical	SF Mental
DASH Total	—	0.412*	0.360*	0.238	−0.014
DASH Work		—	0.683**	0.381*	−0.091
DASH Sports and Music			—	−0.022	0.010
SF Physical				—	0.062
SF Mental					—

Spearman's rho. *p < 0.05; **p < 0.01. Sample size varies across comparisons due to missing data and the optional nature of DASH modules. Pairwise sample sizes were: DASH total vs. DASH modules (*n* = 35), DASH total vs. SF-12 (*n* = 61), DASH work vs. DASH sport/music (*n* = 24), and SF-12 physical vs. mental components (*n* = 61).

#### 3.2.2 Relationship between DASH and SF-12 scores based on whether the affected limb is the dominant extremity

The Mann–Whitney U test was used to analyze differences in functionality and quality of life according to whether the replanted limb corresponded to the dominant or non-dominant extremity.

No statistically significant differences were observed in total DASH scores or in the optional DASH modules (work and sport/music) between patients with dominant and non-dominant limb involvement.

Similarly, no significant differences were identified in the physical component of health-related quality of life. However, patients with dominant limb injuries reported significantly lower mental quality of life compared to those with non-dominant limb involvement. Detailed statistical results are presented in Table 3.

**Table 3:** Comparison of DASH and SF-12 scores according to dominant versus non-dominant limb involvement.

Group		N	Mean rank	Mann–Whitney U test	Sig. (p)
DASH Total	Affected dominant limb	20	21.88	392.500	0.111
	Affected non dominant limb	31	28.66		
	Total	51			
DASH Work	Affected dominant limb	10	10.50	110.000	0.121
	Affected non dominant limb	16	15.38		
	Total	26			
DASH Sports and music	Affected dominant limb	11	15.27	109.000	0.180
	Affected non dominant limb	15	11.09		
	Total	26			
Physical Component Summary (PCS)	Affected dominant limb	19	22.63	349.000	0.264
	Affected non dominant limb	31	27.26		
	Total	50			
Mental Component Summary (MCS)	Affected dominant limb	19	20.34	392.500	0.047*
	Affected non dominant limb	31	28.66		
	Total	50			

*p < 0.05. Sample size varies because dominance data were unavailable for some participants and optional DASH modules were completed only when applicable. SF-12 subgroup analyses include participants with both dominance and SF-12 data available.

#### 3.2.3 Comparison of functionality and quality of life according to the need for surgical reintervention

The non-parametric Mann–Whitney U test was used to assess differences in upper limb functionality and quality of life between patients who required surgical reintervention after the initial replantation procedure and those who did not.

No statistically significant differences were observed in total DASH scores or in the physical component of health-related quality of life between both groups.

However, patients who required reintervention showed significantly worse functional outcomes in the DASH work and sport/music modules compared to those who did not require additional surgery.

Regarding mental quality of life, patients who required reintervention showed lower mean ranks; however, this difference did not reach statistical significance. Detailed statistical results are presented in Table 4.

**Table 4:** Comparison of DASH and SF-12 scores according to surgical reintervention status.

Group		N	Mean rank	Mann–Whitney U test	Sig. (p)
DASH Total	Required reintervention	29	33.57	418.000	0.397
	No reintervention needed	33	29.68		
	Total	62			
DASH Work	Required reintervention	12	23.92	67.000	0.013*
	No reintervention needed	23	14.91		
	Total	35			
DASH Sports and music	Required reintervention	15	21.97	90.500	0.046*
	No reintervention needed	20	15.03		
	Total	35			
SF Physical	Required reintervention	29	32.76	413.000	0.450
	No reintervention needed	32	29.41		
	Total	61			
SF Mental	Required reintervention	29	26.48	595.000	0.055
	No reintervention needed	32	35.09		
	Total	61			

*p < 0.05.

#### 3.2.4 Comparison of functionality and quality of life according to return to work

A Mann–Whitney U test was conducted to examine differences in functionality and quality of life according to whether patients returned to productive employment following replantation.

Patients who did not return to work showed significantly greater functional disability, reflected in higher total DASH scores and worse outcomes in the DASH work and sport/music modules.

No statistically significant differences were observed in the physical or mental components of health-related quality of life between both groups. Detailed statistical results are presented in Table 5.

**Table 5:** Comparison of DASH and SF-12 scores according to return to work status.

Group		N	Mean rank	Mann–Whitney U test	Sig. (p)
DASH Total	Has returned to productive activity	32	25.67	665.500	0.009*
	Has not returned to productive activity	30	37.72		
	Total	62			
DASH Work	Has returned to productive activity	27	14.91	191.500	< 0.001*
	Has not returned to productive activity	8	28.44		
	Total	35			
DASH Sports and music	Has returned to productive activity	21	12.12	270.500	< 0.001*
	Has not returned to productive activity	14	26.82		
	Total	35			
SF Physical	Has returned to productive activity	32	28.36	548.500	0.210
	Has not returned to productive activity	29	33.91		
	Total	61			
SF Mental	Has returned to productive activity	32	32.64	411.500	0.442
	Has not returned to productive activity	29	29.19		
	Total	61			

*p < 0.05. The DASH work module was completed only by participants who reported engagement in occupational activities.

#### 3.2.5 Association between the need for reoperation and return to productive activity

The association between the need for surgical reoperation and return to productive activity was explored using the Chi-square test.

A statistically significant association was observed between reoperation and return to work, indicating that patients requiring additional surgery showed a lower proportion of return to productive activity at the time of assessment.

The distribution of rehabilitation also differed between groups, with patients who underwent reoperation more frequently requiring rehabilitation. Detailed statistical results are presented in Table 6.

**Table 6:** Association between surgical reoperation and return to work.

Reintervention status	Category	Resumption of work activity	Non resumption of work activity	Total
Required reintervention	Observed count	10	19	29
	Expected count	15	14	29
	Corrected residual	−2.5	2.5	
No reintervention required	Observed count	22	11	33
	Expected count	17	16	33
	Corrected residual	2.5	−2.5	

*χ*^2^(1, *N* = 62) = 6.402, *p* = 0.011.

#### 3.2.6 Association between the need for reoperation and completion of rehabilitation

The association between the need for reoperation following replantation and completion of rehabilitation was analyzed using the Chi-square test.

A statistically significant association was observed between both variables, indicating that patients who required reoperation were more likely to complete rehabilitation programs. Detailed statistical results are presented in Table 7.

**Table 7:** Association between surgical reoperation and completion of rehabilitation.

Reintervention status	Category	With Rehabilitation	Without Rehabilitation	Total
Required reintervention	Observed count	29	0	29
	Expected count	25.7	3.3	29
	Corrected residual	2.6	−2.6	
No Required reintervention	Observed count	26	7	33
	Expected count	29.3	3.7	33
	Corrected residual	−2.6	2.6	

*χ*^2^(1, *N* = 62) = 6.934, *p* = 0.008.

#### 3.2.7 Association between the need for reoperation and functional use of the hand in activities of daily living

The relationship between the need for surgical reoperation and subjective perception of hand functionality in activities of daily living was examined using a contingency table and the Chi-square test. This perception was assessed using item 5 of the Russell test, which evaluates functional use of the hand in daily activities. Response categories included ‘never’, ‘little’, ‘for many activities’, ‘for almost all activities’, and ‘for all activities’, representing increasing levels of perceived functional use.

A statistically significant association was observed between reoperation and perceived functional hand use, indicating that patients who required reoperation reported greater functional limitations in activities of daily living, whereas patients who did not require additional surgery reported better perceived functional recovery. Detailed statistical results are presented in Table 8.

**Table 8:** Association between surgical reoperation and perceived functional use of the hand in activities of daily living based on Russel test item 5.

Perceived functional use of the hand in activities of daily living		Never	Little	For many	For almost all	For all
Required reintervention	Observed count	1	15	4	6	3
	Expected count	0.9	7.5	4.7	8	8
	Corrected residual	0.1	4.4	−0.5	−1.1	−2.8
No Required reintervention	Observed count	1	1	6	11	14
	Expected count	1.1	8.5	5.3	9	9
	Corrected residual	−0.1	−4.4	0.5	1.1	2.8

*χ*^2^(4, *N* = 62) = 21.068, *p* < 0.001.

## 4 Discussion

This study aimed to comprehensively analyze the functional recovery, subjective experience, and quality of life in patients undergoing upper limb replantation. Both clinical and social variables are considered, incorporating validated scales that evaluate objective functionality, the perception of surgical outcomes, and the patient's overall quality of life.

One of the main findings is the correlation between scores on the DASH questionnaire and its optional modules for work and sports/music, suggesting that greater overall disability of the upper limb is associated with a greater impact on occupational and recreational activities. This result is consistent with previous studies showing that DASH is highly sensitive in detecting functional limitations in specific activities requiring precision and strength, particularly in patients with complex trauma (15, 16, 20). However, no significant correlations are found between the DASH and the SF-12 components, which reinforces the idea that localized limb functionality does not always translate into a negative perception of global health, especially when compensatory or adaptive factors are present (13, 21).

Regarding the involvement of the dominant limb, results show that patients with replantation of this extremity report lower mental quality of life, although no significant differences are observed in objective functionality. This finding aligns with research indicating that amputation or injury to the dominant limb has a greater psychological and emotional impact, even when motor function can be partially recovered (14, 22). This underscores the importance of integrating psychological support into the rehabilitation process for these patients (23).

Another relevant finding is that the need for surgical reintervention was not associated with significant differences in total DASH scores or in the physical component of the SF-12, but it was associated with worse results in the specific DASH modules related to work and recreational activities. Patients who required reintervention showed greater functional disability in these more demanding contexts. These results suggest that, although reintervention may preserve limb viability, in the present cross-sectional analysis it was associated with worse functional performance in work-related and recreational tasks. However, due to the cross-sectional design of the study, these findings should be interpreted as associations rather than causal relationships (3, 7).

A particularly significant finding is that, despite all reoperated patients undergoing rehabilitation, they show lower perceived hand functionality in activities of daily living according to the Russell test. This result reveals that rehabilitation, although necessary, does not fully compensate for the negative impact of a second surgical intervention (1, 21, 24). The association between reintervention and limited hand use is evident in the overrepresentation of negative responses (“little,” “for many activities”) and underrepresentation of favorable responses (“for all activities”), highlighting the value of the subjective component in evaluating real functional outcomes (1, 12, 13). Recent studies have also emphasized that patients’ perception of their functional capacity is strongly mediated by the number of complications and the degree of residual dependency (25, 26). These findings also reflect patient-perceived satisfaction with functional outcomes following replantation.

These findings highlight the importance of close multidisciplinary follow-up in patients requiring surgical reintervention, as this subgroup may experience greater functional limitations despite rehabilitation. This subgroup may benefit from longer, interdisciplinary, and individualized intervention plans that include psychological support and occupational guidance (28,29).

On the other hand, this study has limitations, such as the sample size, which, although clinically relevant, limits statistical power for some subgroups. Additionally, the use of self-administered questionnaires may be influenced by individual perception factors, fatigue, or social desirability bias. Lastly, objective measures of strength, range of motion, or specific surgical outcomes are not included, which would have allowed for a more robust triangulation of real functionality. Another limitation relates to the heterogeneity of the anatomical level of replantation, which ranged from single-digit injuries to hand and arm replantations. Functional expectations and rehabilitation trajectories differ substantially across these levels. Because the present sample size did not allow stratified analyses by injury level, the results should be interpreted cautiously, as functional outcomes may be influenced by this variability.

In addition, the analyses were based on bivariate non-parametric tests, and no multivariable models were constructed to adjust for potential confounding factors such as age, limb dominance, level of injury, or exposure to rehabilitation. Therefore, the observed associations should be interpreted cautiously, as they may be influenced by uncontrolled confounders.

Despite these limitations, the results of this study have important practical applications. First, they underscore the need to use combined functional and subjective evaluation tools beyond classical surgical outcomes. Second, they identify reintervention as a functional risk factor that should trigger specific follow-up protocols. The Russell test also provided a descriptive overview of patients’ perceived functional use of the limb in daily activities. However, because this instrument has not been formally validated as a psychometric patient-reported outcome measure (PROM) in this population, the results derived from it should be interpreted with caution and considered exploratory indicators of subjective functional perception rather than definitive measures of clinical outcome.

## 5 Conclusions

Upper limb replantation, beyond its surgical success, poses significant challenges in functional recovery, social reintegration, and patient quality of life. This study demonstrates that the need for surgical reintervention and the inability to return to work are critical factors associated with greater perceived disability, especially in occupational and recreational contexts. Despite the high rate of access to rehabilitation, functional limitations persist in subgroups at higher risk of persistent disability, particularly patients requiring reintervention or not returning to work, highlighting the need for a multidimensional and individualized approach to follow-up. The integration of functional, subjective, and quality of life scales provides a more precise framework for clinical decision-making and the optimization of rehabilitative resources in this patient population.

## Data Availability

The original contributions presented in the study are included in the article/Supplementary Material, further inquiries can be directed to the corresponding author.
